# Antihypertensive Drug and Inner Ear Perfusion: An Otologist’s Point of View

**DOI:** 10.3390/ph2020044

**Published:** 2009-09-11

**Authors:** Antonio Pirodda

**Affiliations:** Department of Specialist Surgical and Anesthesiological Sciences, University of Bologna, via Massarenti 9 – 40138 Bologna, Italy; Email: antonio.pirodda@unibo.it

**Keywords:** inner ear perfusion, hemodinamic imbalance, antihypertensive therapy

## Abstract

A number of labyrinthine disorders with sensorineural hearing loss, vertigo, and tinnitus are known to occur to young people without vascular risk factors, thus being classified as “idiopathic” in the absence of satisfactory explanations; in the last decade, this phenomenon has found a reliable explanation by the adverse effect of a sharp decrease of blood pressure values followed by an abnormal vasomotor regulation. This model may not only be applied to healthy subjects, but even had some confirmation in conditions possibly affecting hemodynamic changes, such as heart failure or treated hypertension. In particular, the results of a recent study on the impact of different antihypertensive therapies, which was analyzed by monitoring the onset or enhancement of tinnitus as a symptom of inner ear sufferance, unequivocally demonstrated an increased prevalence of tinnitus in subjects submitted to more “aggressive” treatments. This seems in agreement with recent observations about the model of fluid homeostasis of the inner ear, and suggests, when possible, to resort to treatments with modulatory effects in order to maintain a steady perfusion to the labyrinth thus protecting its function.

## 1. Introduction

A relatively frequent problem otorhinolaryngologists and audiologists must deal with is represented by the onset of labyrinthine troubles (possibly presenting with sudden sensorineural hearing loss, vertigo, and tinnitus) affecting young people without vascular risk factors. As most of these affections are labelled as “idiopathic”, in the absence of a precise cause, it seemed logical to look at some hemodynamic features, not necessarily of pathologic nature, which could be at the origin of the phenomena: namely, it was hypothesized - and to some extent demonstrated - that hypotension followed by an abnormal vasomotor regulation could generate a transient ischemia and a consequent sufferance of the inner ear [1,2]. Actually, a mild hearing loss affecting the low frequencies was found to be significantly more represented in subjects with no vascular risk factors but classified as having essential hypotension as compared to a control population [3]. On the other hand, hypotension itself without a correlated vasomotor altered reactivity seemed not to be sufficient to provoke inner ear damages [4], whereas the latter are possibly favoured by the known reduced ability for autoregulation of cochlear blood flow with respect to the brain flow [5]. 

This argument could represent a satisfactory explanation for a number of cases of labyrinthine impairment affecting young healthy subjects in absence of other recognizable causes; accordingly, it should be kept in mind, as it could be responsible for a recurrence of this kind of disorder when the underlying conditions are not modified. 

Moreover, if in some cases a particular cardiovascular/autonomic profile can be postulated as a basis for explaining this phenomenon [6,7], analogous conditions of imbalance could derive from an insufficient cardiac activity, possibly represented by heart failure [8] or, in a wider population, from a pharmacologic treatment: this is to be considered when planning an antihypertensive therapy.

## 2. Antihypertensive Therapy and the Inner Ear

To study the possible effect of different antihypertensive drugs on the inner ear function, tinnitus appears as an available symptom. Actually, it is a non-specific disturbance which can derive from any level of the auditory pathways, but implies a cochlear involvement in a number of cases [9]: this seems particularly reasonable when considering cases of recent arousal or cases of intermittence or variations in intensity of the symptom. On this basis, an investigation firstly concerning the prevalence of tinnitus in a population of patients with hypertension, and secondly analyzing the impact of different antihypertensive drugs on the incidence of this symptom in these patients was made [10]. As a matter of fact, the possibility of generating tinnitus is well known for the most widely used antihypertensive drugs [11]: however, it seemed of some interest to investigate on the possible correlations between tinnitus and drug activity. Briefly, it was found that the prevalence of tinnitus was relatively high (17.6%) in the whole group, and mostly that the subgroup treated with diuretics presented a significant higher incidence of tinnitus (27.2%) compared with the subgroups under treatment with angiotensin II receptor blockers (13.5%), alfa-blockers (21.8%), HMG CoA reductase inhibitors (12.3%). Moreover, the onset of tinnitus was associated with a dramatic decrease in systolic blood pressure that could be related to the peak effect of the pharmacological treatment in 11.9% of the patients who presented this symptom [10]. 

Even if the study did not investigate any causal factor(s) of tinnitus, which can even present as a symptom deriving from an underlying vascular damage, the reported data seem to confirm a possible involvement of a rapid lowering of blood pressure values, followed by a sharp vasoconstriction, among the threatening factors to the labyrinth. It must be considered that the latter is characterized by a blood supply of terminal type, and therefore represents a typical model of end-organ which can be more easily prone to damages linked to an acute lack of perfusion. In addition, the complexity of the labyrinthine structures is to be considered, as a noteworthy amount of energy is required in order to maintain the homeostasis of fluids: this can result in an overexposure of the inner ear to adverse effects due to hemodynamic variations.

Indirect support for this interpretation is provided by the recent report of a therapeutic effect of water intake in Meniere Disease [12]: this labyrinthine disorder, characterized by crises of fluctuating hearing loss, tinnitus and vertigo, is generally attributed to an endolymphatic hydrops although after several decades it has not yet reached a precise pathogenic definition. Despite these uncertainties, it has been widely treated with diuretic therapy without a completely exhaustive rationale: conversely, the possibility to obtain a cure by water is explained by a positive effect of the reduced plasma osmolality on the modulation of secretion and action of vasopressin [12], which results in a more satisfactory control of the hemodynamic balance. 

As already mentioned, the latter seems to be a not negligible factor for maintaining the regulation of the inner ear fluids, which derives from complex processes recently better clarified by focusing on the presence and the role of aquaporins [13]: these proteins, that even increase the water permeability in the kidney loop of Henle, represent both a solely osmotic, hormone-independent mechanism of water reabsorption and a hormone-dependent one linked to the activity of vasopressin, and are considered essential for labyrinthine homeostasis [13]. The observation that vasopressin application leads to endolymphatic hydrops in vivo [14], depending on an enhancement of the water influx into the endolymph compartment through an increase of aquaporin water channels [13], is a further confirmation of the influence of both osmolality and hormonal interference on the inner ear, and indirectly outlines the importance of maintaining a steady perfusion to this organ: to this purpose, the same considerations which strongly suggest to avoid renal hypoperfusion are reliable; as a matter of fact, actually, important analogies exist between the kidney and the inner ear concerning the functional mechanisms of water and ion regulation [13], which can even be considered the molecular basis for unwanted side effects of diuretics and allow a parallelism between ototoxicity and nephrotoxicity [15].

These observations, along with the results concerning our group of patients with sudden sensorineural hearing loss [1,2] are in agreement of a classical experimental setting of Maass [16] concerning an animal model of hemorrhagic hypotension, which showed decreased intracochlear PO2 values in proportion to the hypovolemic hypotension and associated to vasoconstriction due to an increase of sympathetic drive. 

In conclusion, it must be firstly precised that the present paper does not aim at discussing about the primary necessity of setting a safe and effective therapy in subjects with hypertension, which obviously must be applied according to the most appropriate modalities in order to avoid major cardiovascular events; moreover, further prospective studies are needed to draw precise conclusions about the relationship between tinnitus and antihypertensive drugs. However, the awareness of some aspects concerning the effects on a peripheral sensory organ could be of some additional utility when choosing the therapy; from this point of view, it should be considered that drugs that block the vascular effect of either sympathetic activation, as alfa-blockers, or tissue renin – angiotensin system, as angiotensin II receptor blockers, seem to be useful to reduce tinnitus [10], on one hand, and that a rapid reduction of the volemia, deriving e.g. from diuretics, could result in a threaten to the labyrinth, on the other hand. 
